# The many faces of cytokine storm syndrome: immunopathogenic mechanisms and clinical implications for a better patient management

**DOI:** 10.1093/cei/uxag039

**Published:** 2026-07-02

**Authors:** Piero Ruscitti

**Affiliations:** Department of Biotechnological and Applied Clinical Sciences, University of L'Aquila, L'Aquila, Italy

**Keywords:** cytokine storm syndrome, inflammation, hyperferritinemia, prognosis

## Abstract

Cytokine storm syndrome is a life-threatening hyperinflammatory condition characterized by excessive and dysregulated immune activation, leading to multiorgan dysfunction and poor clinical outcomes if not promptly recognized and adequately treated. Rather than representing a single disease entity, cytokine storm syndrome is increasingly understood as a final common pathogenic pathway shared by a broad spectrum of clinical conditions converging on overlapping immunopathological mechanisms driven by sustained cytokine production, immune-cell hyperactivation, and failure of immune-regulatory pathways. The clinical presentation of cytokine storm syndrome is characterized by a rapidly progressive nature, multisystem involvement, and the diagnostic challenges arising from nonspecific symptoms and overlapping laboratory features. Hyperferritinaemia emerges as a central laboratory hallmark with both diagnostic and potential pathogenic relevance. Therapeutic management of cytokine storm syndrome requires early recognition and is guided by three core principles: supportive care for organ dysfunction, control of underlying triggers, and timely immunomodulatory or immunosuppressive interventions. Overall, cytokine storm syndrome represents a complex and heterogeneous clinical syndrome in which improved mechanistic understanding, biomarker development, and tailored therapeutic strategies are essential to optimize patient outcomes.

## Teaser

Cytokine storm syndrome is a life-threatening hyperinflammatory condition characterized by excessive and dysregulated immune activation, leading to multiorgan dysfunction and poor clinical outcomes if not promptly recognized and adequately treated. Rather than representing a single disease entity, cytokine storm syndrome is increasingly understood as a final common pathogenic pathway shared by a broad spectrum of clinical conditions converging on overlapping immunopathological mechanisms driven by sustained cytokine production, immune-cell hyperactivation, and failure of immune-regulatory pathways. The clinical presentation of cytokine storm syndrome is characterized by rapidly progressive nature, multisystem involvement, and the diagnostic challenges arising from nonspecific symptoms and overlapping laboratory features.

## Introduction, concept and definition of cytokine storm syndrome

**Figure 1:**
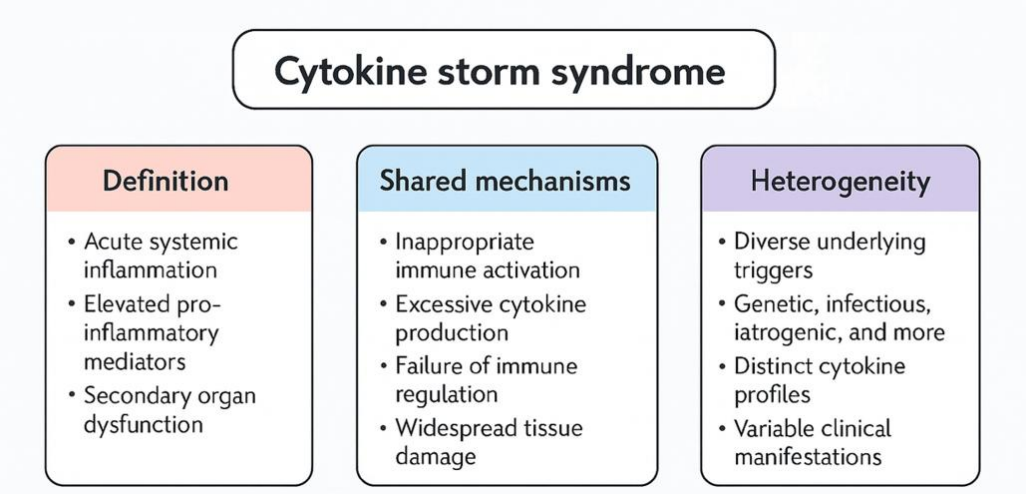
Distinct aetiological conditions, including monogenic disorders, infectious diseases, rheumatologic disorders, and malignancies, converge towards a shared hyperinflammatory state leading to cytokine storm syndrome. Despite heterogeneous triggers, the diagnosis relies on a common set of integrated criteria comprising persistent high-grade fever, sustained elevation of pro-inflammatory mediators, and secondary organ dysfunction.

An increasing body of evidence has recently established the cytokine storm syndrome as a life-threatening hyperinflammatory condition that, if not promptly recognized and appropriately managed, may rapidly progress to multiorgan dysfunction and poor clinical outcomes [1, 2]. Importantly, cytokine storm syndrome is no longer regarded as a single disease entity. Instead, it is currently conceptualized as a final common immunopathological pathway resulting from profound dysregulation and amplification of immune responses arising in diverse clinical settings [3, 4]. In fact, cytokine storm syndrome encompasses a broad and expanding spectrum of hyperinflammatory states associated with heterogeneous aetiologies, including monogenic disorders, infectious diseases, rheumatologic disorders, and malignancies [1–4]. In parallel, growing attention has been directed towards iatrogenic forms of cytokine storm syndrome, particularly in the setting of more recent immunotherapeutic strategies, such as chimeric antigen receptor T-cell therapies, in which the syndrome is commonly designated as cytokine release syndrome [5, 6]. These observations may underscore the clinical and biological heterogeneity inherent to this condition, as reported in Fig. 1. To provide a more robust and clinically meaningful definition of cytokine storm syndrome, recent conceptual and clinical outlines have emphasized the necessity of a multidimensional approach integrating clinical manifestations, laboratory abnormalities, and underlying pathophysiological mechanisms [7]. Rather than being defined by a single biomarker or isolated clinical feature, cytokine storm syndrome is increasingly characterized by the concurrent presence of multiple defining elements [7]. Central to this context is the emergence of an acute systemic inflammatory clinical scenario, typically manifested by persistent high-grade fever and constitutional symptoms, together with a marked and sustained elevation of circulating pro-inflammatory mediators, ultimately evolving to secondary organ dysfunction [7]. By incorporating clinical presentation, cytokine excess, and organ dysfunction into a unified definition, this approach may facilitate a more consistent strategy for diagnosis, risk stratification, and therapeutic decision-making, while simultaneously acknowledging the diversity of initiating triggers and underlying possible different diseases [3, 4, 7, 8]. Also, this integrative conceptualization underscores that cytokine storm syndrome represents not merely an exaggerated inflammatory response but rather a qualitative breakdown of immune homeostasis driven by possible diverse underlying aetiologies [8].

## Pathogenic and immunological mechanisms

From a pathogenic standpoint, the development of cytokine storm syndrome reflects the convergence of many non-mutually exclusive immunopathological mechanisms, including inappropriate immune triggering or aberrant danger sensing, an excessive magnitude and duration of immune activation, and/or failure of the regulatory and inhibitory pathways responsible for the restoration of immune homeostasis [3, 4, 7, 8]. These pathways predominantly involve aberrant activation of macrophages, qualitative and quantitative dysfunction of T-lymphocyte responses, and exaggerated signalling by innate immune sensing systems [7–9]. Within this intricate mechanistic framework, cytokine production becomes more and more quantitatively disproportionate to the initiating stimulus and progressively uncoupled from effective pathogen elimination or tissue repair [9]. Consequently, cytokines cease to function as mediators of controlled host defence and instead act as potent and uncontrolled drivers of sustained immune response [9, 10]. Under normal physiological conditions, inflammatory responses are tightly regulated by a finely balanced interplay between activating and suppressive mechanisms. These include the induction and functional integrity of regulatory immune cell populations, the production of anti-inflammatory mediators, adaptive metabolic reprogramming of immune cells, and the engagement of specialized resolution pathways that actively terminate inflammatory responses once the inciting stimulus has been neutralized. In the context of cytokine storm syndrome, this counter-regulatory regulatory architecture may become quantitatively insufficient or functionally ineffective [9–11]. Taken together, as a consequence of this sustained overwhelming immune response, the development of multiorgan dysfunction may occur, reflecting a process of immune-mediated collateral damage in association with tissue hypoperfusion or direct cytotoxic effects [7].

## Aetiology-specific pathogenic pathways

Despite distinct underlying aetiologies and possible differences in predominant pathogenic mechanisms, a convergence towards a shared hyperinflammatory phenotype is observed during the cytokine storm syndrome. In this context, the aberrant immune recognition is classically illustrated by hypersensitivity reactions, in which disproportionate immune responses are elicited [3, 7]. Conversely, defective immune recognition in conjunction with immune evasion is a defining feature of Epstein–Barr virus-associated haemophagocytic lymphohistiocytosis (HLH) [12–14], wherein impaired cytotoxic lymphocyte function results in persistent antigenic stimulation and uncontrolled macrophage activation [15, 16]. In this context, histological evidence of haemophagocytosis, most commonly detected in the bone marrow, should be regarded as a manifestation of excessive macrophage activation rather than as a disease-specific or pathognomonic finding [16]. HLH is usually classified into primary and secondary forms [17]. Primary HLH is caused by inherited defects affecting cytotoxic granule exocytosis and lymphocyte-mediated cytotoxicity, whereas secondary HLH develops in response to external triggers, including infections, malignant disorders, and autoimmune or autoinflammatory diseases [17]. In the spectrum of secondary HLH, macrophage activation syndrome (MAS) represents a prototypical clinical entity associated with rheumatic diseases, particularly during Still's disease. The occurrence of HLH and associated cytokine storm syndrome is sustained by persistent antigen stimulation combined with defective downregulation of inflammatory pathways, leading to persistently deregulated overwhelming immune response [15–17].

In contrast, iatrogenic forms of cytokine storm syndrome, most notably those occurring after chimeric antigen receptor T-cell therapy, are characterized by pronounced effector immune activation and massive cytokine release driven by rapid T-cell expansion and secondary activation of monocytes and macrophages [18]. This paradigm exemplifies a therapeutically induced form of immune hyperactivation and highlights the potential for specific targeted immunomodulatory strategies to effectively attenuate downstream inflammatory cascades.

During sepsis, a paradoxical coexistence of immune evasion and hyperinflammation is frequently observed [14]. In fact, invading pathogens may evade effective immune clearance in association with a profoundly dysregulated host immune response, failing to appropriately terminate inflammation [14].

## Cytokine profiles and immune heterogeneity

Notwithstanding the diversity of initiating events, the heterogeneous aetiologies of cytokine storm syndrome converge on a restricted set of shared downstream pathogenic pathways [19, 20]. Within this unifying framework, however, an additional level of complexity is introduced by the substantial heterogeneity of cytokine profiles observed among distinct underlying diseases [20–22]. In fact, the preferential dominance of specific cytokines could instead reflect the cellular architecture, immune checkpoint regulation, and intracellular signalling networks that are specifically engaged within each disease pathogenesis [23].

In the context of Still's disease and its life-threatening evolution, interleukin-1 (IL-1) and IL-18 emerge as central and interconnected pathogenic mediators that orchestrate the hyperinflammatory response [24, 25]. Both cytokines belong to the IL-1 family and are primarily produced by activated innate immune cells, particularly monocytes and macrophages, following inflammasome activation. Their sustained release reflects a profound dysregulation of innate immune sensing and cytokine control mechanisms [24, 25].

In the context of primary and secondary HLH, interferon-γ (IFN-γ) emerges as a central pathogenic mediator, consistent with impaired cytotoxic lymphocyte activity and persistent antigenic stimulation [26]. IFN-γ is able to sustain macrophage activation, to promote haemophagocytosis, to enhance antigen-presenting capacity, and to amplify immune-mediated tissue injury, thereby functioning as a principal regulator of the HLH-associated inflammatory pathways [26, 27].

In contrast, in therapy-related cytokine release syndromes, the inflammatory milieu is primarily related to IL-6. This cytokine is a key mediator of the acute-phase response and exerts pleiotropic effects on endothelial activation, pyrexia, and vascular permeability [28]. Its predominance reflects a pathogenic circuitry in which innate immune activation, especially involving monocytes and macrophages, plays a dominant role, with secondary consequences on coagulation pathways, metabolic regulation, and vascular homeostasis [29].

Tumour necrosis factor (TNF), while not uniformly predominant across cytokine storm syndrome subtypes, represents a pleiotropic and context-dependent mediator within the hyperinflammatory landscape [30]. Depending on temporal dynamics and tissue-specific expression, TNF may also contribute to the amplification of parallel cytokine signalling pathways or act synergistically with IFN-γ or IL-1 to exacerbate immune-mediated damage [31]. Accordingly, the relative contribution of TNF appears to modulate patterns of organ involvement and disease severity rather than to delineate a distinct cytokine storm syndrome subtype.

This variability at the cytokine level may highlight relevant clinical, diagnostic, and therapeutic implications. It supports the interpretation of the cytokine storm syndrome as a heterogeneous condition characterized by disease-specific immune signatures. The latter may provide mechanistic points for a better understanding of the variability observed in clinical phenotypes among patients with cytokine storm syndrome arising from different underlying aetiologies [3, 7, 32].

## Influence of age and immunosenescence

An additional possible pathogenic dimension of cytokine storm syndrome concerns the influence of ageing on immune regulation [33]. This clinical aspect remains insufficiently explored so far, and it warrants dedicated, specifically designed studies. In fact, although cytokine storm syndrome is regarded as an age-independent hyperinflammatory state, growing evidence suggests that ageing may be able to modify the immunological milieu, thereby possibly affecting the occurrence of this condition [33]. Age-associated inflammaging, sustained by the senescence-associated secretory phenotype, lowers the threshold for immune dysregulation, while qualitative defects in innate and adaptive immunity favour persistent cytokine amplification and impaired resolution [33]. Concurrently, reduced physiological reserve and organ resilience amplify the impact of cytokine-mediated injury, potentially leading to earlier and less reversible organ dysfunction [34, 35]. Despite the possible biological plausibility, the contribution of ageing to cytokine storm syndrome remains insufficiently defined, needing further studies to be fully clarified.

## Clinical presentation and disease evolution

**Figure 2:**
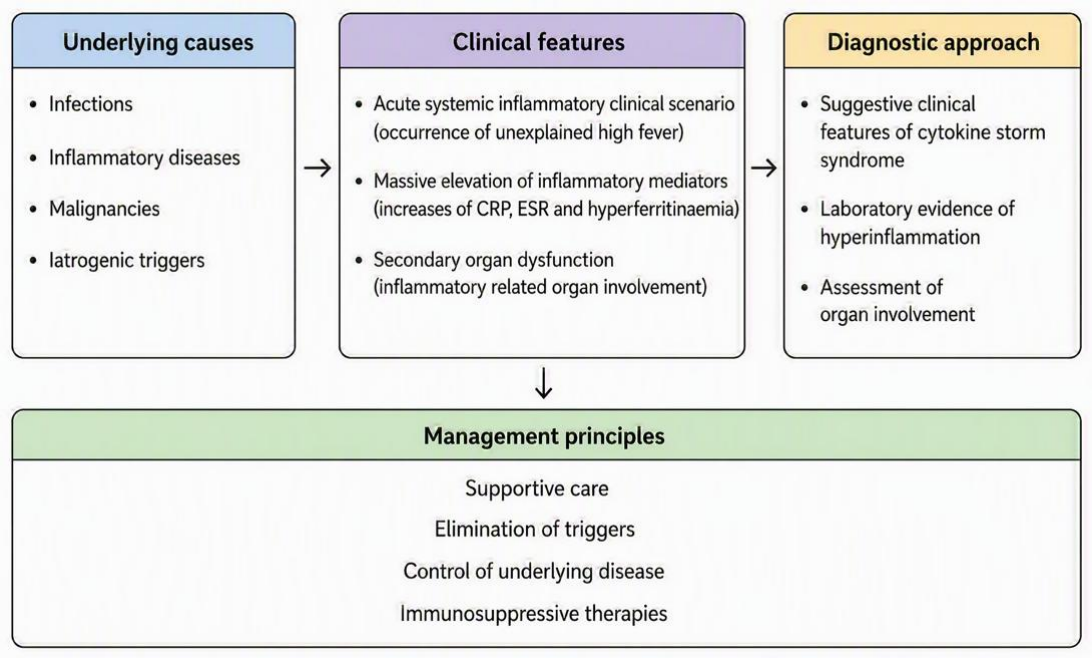
In this figure, a clinical algorithm is proposed to approach the cytokine storm syndrome, comprising all aetiologies. Firstly, different aetiologies are recognized, the suspicion of the occurrence of cytokine storm syndrome should raise in patients with unexplained fever, massive increases of inflammatory markers together with hyperferritinaemia, and multi-organ failure. Once the diagnosis is made, according to the simultaneous presence of the simultaneous coexistence of the occurrence of an acute systemic inflammatory clinical scenario associated with massive elevation of pro-inflammatory mediators and secondary organ dysfunction, the management should comprise the supportive care to maintain the organ function, the control of the underlying disease and/or elimination of triggers, and the immunosuppression to limit the collateral damage of the aberrant activation of the immune system.

From a clinical perspective, cytokine storm syndrome represents a rapidly evolving inflammatory condition characterized by a progression from systemic inflammation to life-threatening multiorgan failure [36]. Unlike inflammatory or infectious conditions with organ-specific or stepwise evolution, cytokine storm syndrome is distinguished by an early loss of anatomical and physiological compartmentalization of inflammation, resulting in near-simultaneous multisystem involvement [3, 7]. This intrinsic feature renders clinical assessment particularly challenging and necessitates continuous reassessment and anticipatory management, as reported in Fig. 2 [37].

Although cytokine storm syndrome may arise from heterogeneous aetiologies, the clinical phenotype may converge due to cytokine amplification independently of the initiating trigger, but maintaining some characteristics of the underlying disease. Consequently, early clinical manifestations often provide limited discrimination regarding the underpinning cause [38–40]. Persistent high-grade fever represents a defining early feature of cytokine storm syndrome and is typically the first clinically evident sign [1–4]. Driven by sustained cytokine-mediated activation of central thermoregulatory pathways, fever in cytokine storm syndrome is frequently refractory to standard antipyretic drugs and closely mirrors the intensity and persistence of immune activation [7, 8]. Accordingly, fever should be regarded not merely as a symptom but as a biological indicator of ongoing excessive production of cytokines. Constitutional manifestations, including severe fatigue, anorexia, and malaise, are nearly universal and often precede overt organ dysfunction [38–40]. Progression of cytokine storm syndrome is marked by rapid multisystem involvement. Cutaneous, gastrointestinal, and musculoskeletal manifestations reflect immune-mediated vascular and tissue inflammation, while involvement of the central nervous system, ranging from cognitive dysfunction to delirium or seizures, constitutes a particularly ominous indicator of disease severity [38–40]. The most severe clinical evolution of cytokine storm syndrome culminates in acute multiorgan failure, encompassing coagulopathy with thrombotic and haemorrhagic features, acute respiratory distress syndrome, acute kidney injury, liver dysfunction, and cardiovascular collapse [38–40]. These manifestations primarily reflect widespread endothelial activation, dysregulated coagulation, and immune-mediated tissue injury. Pulmonary involvement is particularly frequent and often necessitates advanced respiratory support, marking a critical inflection point in prognosis [3, 7]. These manifestations may be variably more represented according to underlying disease, and they are reported in Table 1.

**Table 1:** Clinical scenarios of cytokine storm syndrome according to different underlying diseases.

Primary and secondary HLH	Sepsis	Cytokine release syndrome
*Triggers/risk factors*		
Infections, rheumatologic conditions, malignancies, genetic conditions	Uncontrolled infections, immunodeficiencies	Immunotherapies, chemotherapy drugs, severe infections
*Dominant cytokine profile*		
IL-1, IL-18, IFN-γ	IL-1	IL-6
*Clinical manifestations*		
Fever and multiorgan dysfunction	Fever and multiorgan dysfunction	Fever and multiorgan dysfunction
Hepatosplenomegaly	Hypotension	Hypotension
Hepatobiliary dysfunction	Hypoxia	Hypoxia
Lymphadenopathies	ARDS	Capillary leak syndrome
Neurologic features	Cardiomyopathy	Diffuse intravascular coagulation
Coagulopathy	Thrombosis	Renal involvement
*Laboratory markers*		
High CRP and ferritin	High CRP and ferritin	High CRP and ferritin
Paradoxically low ESR	High ESR	High ESR
Cytopenias	High procalcitonin in bacteriemia	Cytopenias
High triglycerides	High neutrophil count	High creatinine
High transaminases	High transaminases	High transaminases
*Main treatment principles*		
Supportive care	Supportive care	Supportive care
Elimination of trigger	Elimination of trigger	Elimination of trigger
Corticosteroids	Corticosteroids	Corticosteroids
Cyclosporin A	Antibiotics	IL-6 inhibitor
IL-1 and IFN-γ inhibitors	IVIGs	Antithymocyte globulin
JAK inhibitors	IL-1 inhibitor	JAK inhibitors

Taken together, the rapid escalation, phenotypic overlap with sepsis and inflammatory flares, and potential for irreversible organ injury render early recognition of cytokine storm syndrome inherently difficult yet critically important. Maintaining a high index of suspicion in patients with persistent fever, escalating inflammatory markers, and evolving multisystem involvement is essential and of clinical relevance. Ultimately, timely identification and prompt intervention remain the principal determinants of outcome in cytokine storm syndrome, underscoring the importance of recognizing its characteristic clinical trajectory.

## Diagnostic challenges and laboratory features

The diagnosis of cytokine storm syndrome remains particularly complex [7]. Early manifestations such as fever, constitutional symptoms, and evolving organ dysfunction are common to a broad spectrum of inflammatory and non-inflammatory disorders, frequently resulting in delayed recognition of cytokine storm syndrome until the syndrome has progressed to advanced stages. Consequently, reliance on clinical features alone is insufficient, underscoring the central importance of laboratory assessment in supporting diagnosis and risk stratification.

Laboratory abnormalities in cytokine storm syndrome reflect the magnitude and persistence of systemic immune activation and generally correlate with disease severity [1–4]. Typical findings include marked elevation of inflammatory markers, hypertriglyceridaemia, increased D-dimer concentrations, cytopenias, and qualitative or quantitative leukocyte abnormalities. These alterations arise from cytokine-driven bone marrow dysregulation, immune-mediated cellular consumption, endothelial activation, and deranged immunothrombotic pathways [7]. Coagulation abnormalities further reflect widespread endothelial injury, whereas cytopenias may be associated with a profound immune activation or evolving haemophagocytic processes.

Among available laboratory parameters, although specific biomarkers are not available yet, hyperferritinaemia has emerged as an informative feature of cytokine storm syndrome, even if it should be carefully contextualized [41–43]. Markedly elevated ferritin levels may frequently precede overt multiorgan failure and may assist in distinguishing cytokine storm syndrome from other inflammatory states [42, 43]. In addition, increasing experimental and clinical evidence may also support the concept that ferritin should not be regarded solely as an epiphenomenon of inflammation, but rather as an active participant in disease pathogenesis [41]. In fact, the hyperferritinaemia may potentially contribute to immune amplification, oxidative stress, and perpetuation of inflammatory cascades according to the concept of the ‘hyperferritinaemic syndrome’ [40, 41].

In the absence of a single pathognomonic test, contemporary diagnostic approaches increasingly emphasize integrative and multidimensional frameworks. The combined assessment of clinical features, hyperferritinaemia, and selected cytokine profiles may enhance diagnostic accuracy and facilitate the identification of specific cytokine storm syndrome subtypes. This approach may be particularly relevant in MAS, in which integration of ferritin levels with cytokines, such as IFN-γ and IL-10, together with evidence of multisystem involvement [44], may help in differentiating the occurrence of cytokine storm syndrome and patients with life-threatening evolution, consequently guiding possible early therapeutic decision-making.

Overall, the diagnostic evaluation of cytokine storm syndrome requires a high index of suspicion and a structured approach integrating clinical assessment with laboratory and immunological data [45]. Despite advances in biomarker characterization, the absence of standardized diagnostic criteria and the limited availability of rapid cytokine assays remain significant limitations. Further efforts aimed at validating biomarker panels and incorporating them into pragmatic diagnostic algorithms are essential to improve early recognition and clinical outcomes in patients with cytokine storm syndrome [46–48].

## Therapeutic principles and management strategies

Timely recognition of cytokine storm syndrome is essential, as the temporal relationship between disease onset and therapeutic intervention represents a critical determinant of clinical outcome [49–51]. Owing to the rapidly progressive and self-reinforcing nature of the inflammatory cascade, delays in treatment initiation are frequently associated with irreversible organ injury and a substantial increase in mortality. Consequently, early management is often unavoidably empirical and must be informed by pathophysiological considerations rather than definitive aetiological confirmation [51]. Current therapeutic strategies are broadly organized around three interdependent principles: (i) delivery of comprehensive supportive care, (ii) identification and control of the inciting trigger, and (iii) prompt initiation of immunosuppressive or immunomodulatory interventions aimed at limiting immune-mediated tissue injury [49–51].

Supportive care constitutes the immediate foundation of management across the full spectrum of cytokine storm syndrome. Given the high likelihood of rapid clinical deterioration, admission to a high-dependency or intensive care setting is frequently required [52]. Supportive measures include haemodynamic stabilization, respiratory support ranging from non-invasive supplementation to invasive mechanical ventilation, renal replacement therapy when indicated, correction of coagulation abnormalities, and judicious fluid management, particularly in the presence of capillary leak [52]. Importantly, supportive interventions should be implemented concomitantly with disease-directed immunomodulatory therapy, as organ support alone does not modify the underlying inflammatory process [52, 53].

Concomitant with supportive management, prompt identification and treatment of the precipitating condition remains a necessary component of care. However, in cytokine storm syndrome, control of the initiating trigger is rarely sufficient as an isolated intervention, since the inflammatory cascade frequently becomes functionally autonomous once established. Accordingly, aetiologically targeted therapy should be combined with direct modulation of the dysregulated immune response [52–54].

Systemic glucocorticoids remain the cornerstone of pharmacological management across cytokine storm syndrome subtypes, demonstrating broad efficacy irrespective of underlying aetiology [55, 56]. Their pleiotropic actions include inhibition of pro-inflammatory cytokine transcription, suppression of immune-cell activation, stabilization of endothelial integrity, and attenuation of capillary leak. The choice of dose, route, and duration should be individualized according to disease severity, rate of progression, and patient-specific factors. Initiation of glucocorticoid therapy at an early stage, ideally prior to the development of irreversible organ dysfunction, is consistently associated with improved outcomes.

In selected clinical contexts, early administration of anti-IL-1 agents or intravenous immunoglobulins may provide effective immunomodulation while limiting broad immunosuppression [57, 58]. These approaches are particularly relevant in the early or evolving phases of cytokine storm syndrome, as they may disrupt inflammatory amplification pathways without substantially impairing host defence mechanisms or confounding diagnostic evaluation [49–51]. Their overall safety profile supports consideration even in the absence of definitive aetiological classification in patients with rapidly progressive disease [57, 58].

In cases characterized by refractory inflammation or progressive organ dysfunction despite first-line therapy, escalation strategies are warranted. These include cytotoxic or T-cell–directed agents, such as etoposide and cyclosporine A, which are of particular relevance in HLH–spectrum disorders [51]. In parallel, targeted inhibition of IFN-γ has emerged as a rational and effective therapeutic strategy in selected HLH contexts, directly addressing a central driver of macrophage activation and immune-mediated tissue injury [59–61].

For refractory, relapsing, or rapidly progressive cytokine storm syndrome, Janus kinase (JAK) inhibitors represent an emerging therapeutic option, providing broader suppression of cytokine-dependent signalling pathways downstream of multiple inflammatory mediators [62, 63]. Their use reflects a shift towards pathway-oriented rather than single-cytokine–directed intervention. Nevertheless, careful patient selection is required, particularly in individuals with advanced age or significant comorbidity burden, given the associated risks of infection and thromboembolic complications.

Across all therapeutic modalities, secondary infection represents a major and persistent concern. Immunosuppressive therapies should therefore be accompanied by close infectious surveillance and, when appropriate, prophylactic antimicrobial strategies, particularly in patients receiving prolonged or combined therapies. Given the complexity of decision-making, a multidisciplinary approach involving intensivists, haematologists, rheumatologists, infectious disease specialists, and immunologists is frequently required [49–51].

Taken together, effective management of cytokine storm syndrome depends on early recognition, rapid initiation of supportive and immunomodulatory therapy, and continuous reassessment of disease trajectory and treatment-related complications. A flexible, mechanism-oriented strategy, tailored to disease severity, dominant inflammatory pathways, and patient-specific vulnerability, offers the greatest potential for interrupting the hyperinflammatory cascade and improving clinical outcomes in this severe and life-threatening syndrome.

## Conclusions

Cytokine storm syndrome constitutes a clinically heterogeneous yet pathophysiologically convergent syndrome, in which diverse aetiological triggers and immune drivers culminate in an overlapping hyperinflammatory phenotype. Within this framework, timely recognition, rigorous clinical and laboratory assessment, and the implementation of appropriately tailored immunomodulatory strategies remain pivotal determinants of patient outcome. Although currently available diagnostic biomarkers are imperfect and context-dependent, early therapeutic intervention, addressing both the precipitating insult and the ensuing immune dysregulation, is essential to mitigate immune-mediated tissue injury and avert irreversible organ dysfunction. Ongoing progress in the understanding and management of cytokine storm syndrome will depend on coordinated translational and clinical research efforts aimed at refining diagnostic frameworks, validating robust and accessible biomarkers, and optimizing therapeutic strategies across the diverse clinical contexts in which this syndrome arises. The integration of mechanistic insight with pragmatic, mechanism-informed clinical approaches represents a critical step towards improving outcomes across the many clinical expressions of cytokine storm syndrome.

## Data Availability

Not applicable, this is a review of published evidence.
